# Supplementary figures and images for: Personality, subjective well-being, and the serotonin 1a receptor gene in common marmosets (Callithrix jacchus)
Source: PLoS One. 2021 Aug 9;16(8):e0238663. doi: 10.1371/journal.pone.0238663 (PMC8351977; doi:10.1371/journal.pone.0238663)

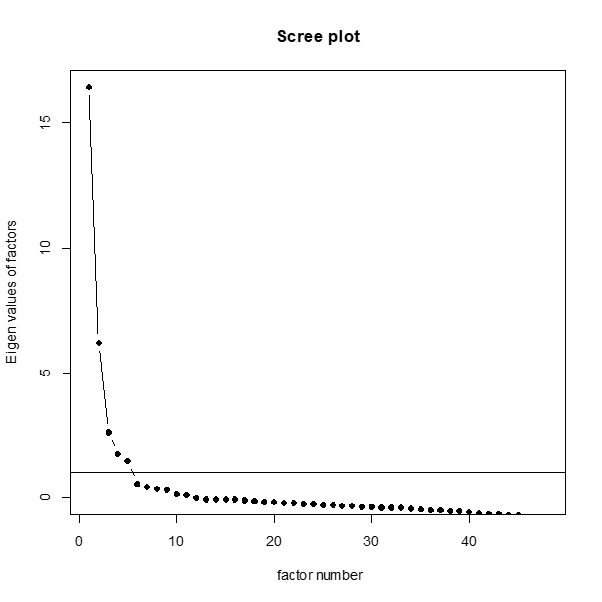

Supplement: S1 Fig — (TIF) [file pone.0238663.s001.tif]

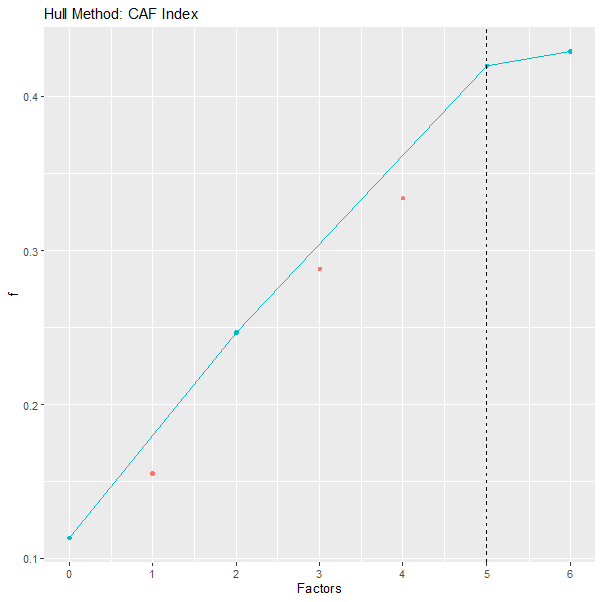

Supplement: S2 Fig — (TIF) [file pone.0238663.s002.tif]

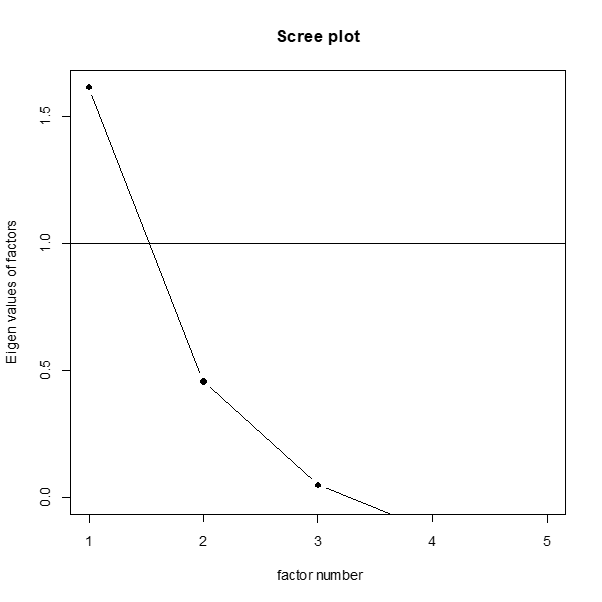

Supplement: S3 Fig — (TIF) [file pone.0238663.s003.tif]

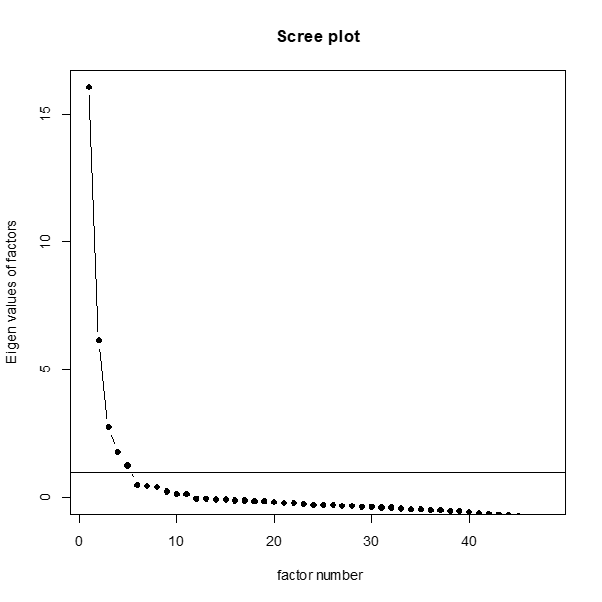

Supplement: S4 Fig — (TIF) [file pone.0238663.s004.tif]

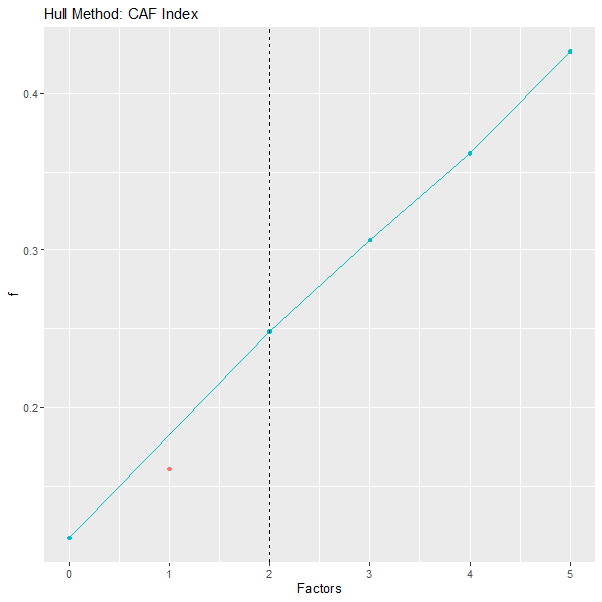

Supplement: S5 Fig — (TIF) [file pone.0238663.s005.tif]

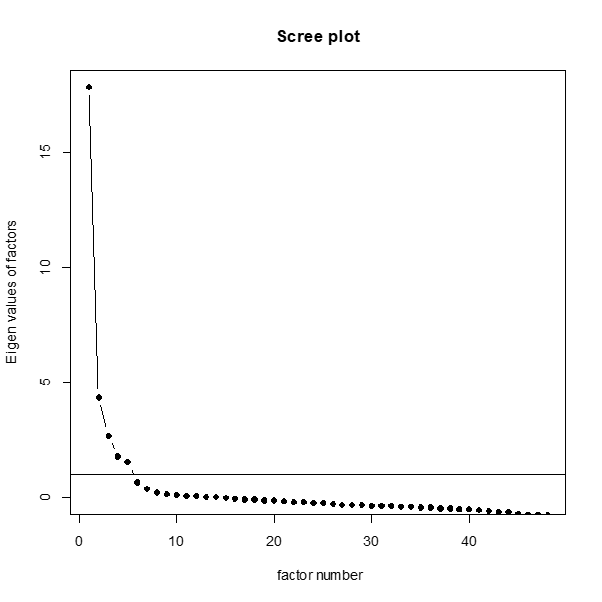

Supplement: S6 Fig — (TIF) [file pone.0238663.s006.tif]

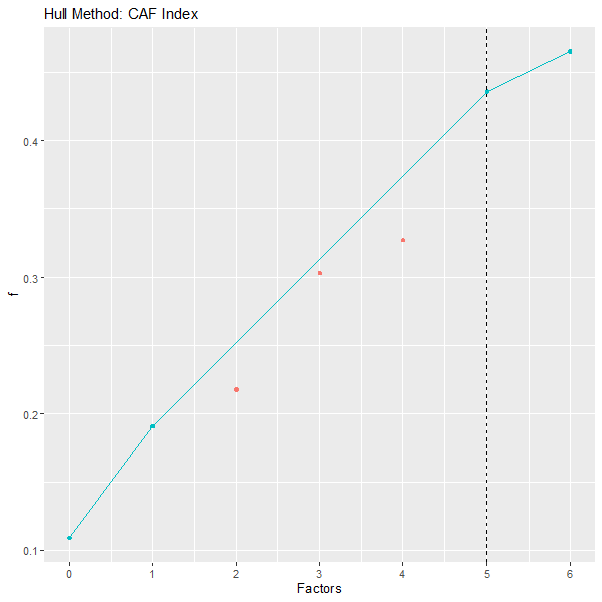

Supplement: S7 Fig — (TIF) [file pone.0238663.s007.tif]

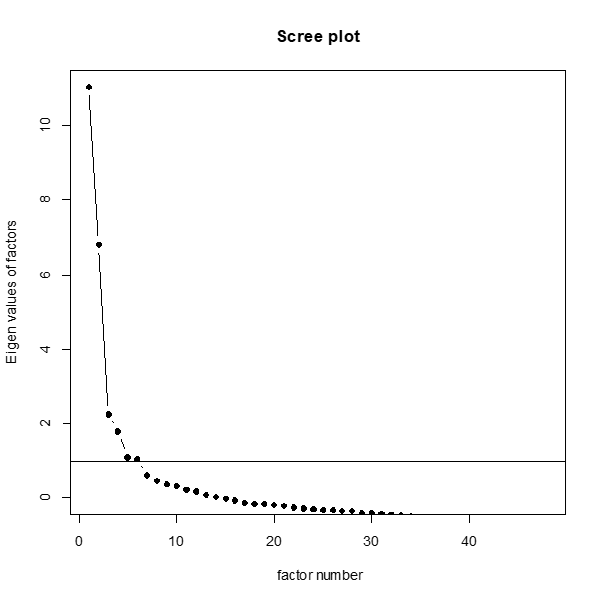

Supplement: S8 Fig — (TIF) [file pone.0238663.s008.tif]

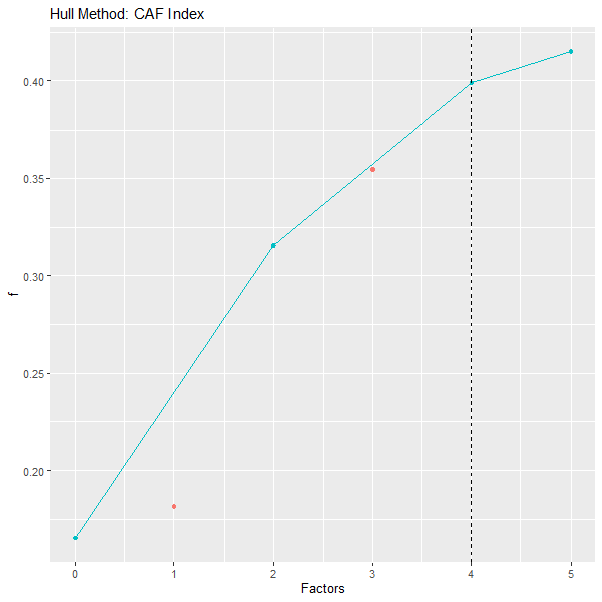

Supplement: S9 Fig — (TIF) [file pone.0238663.s009.tif]

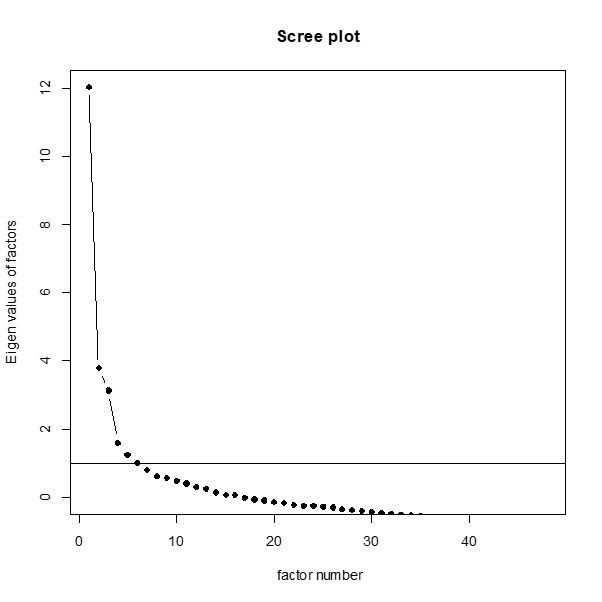

Supplement: S10 Fig — (TIF) [file pone.0238663.s010.tif]

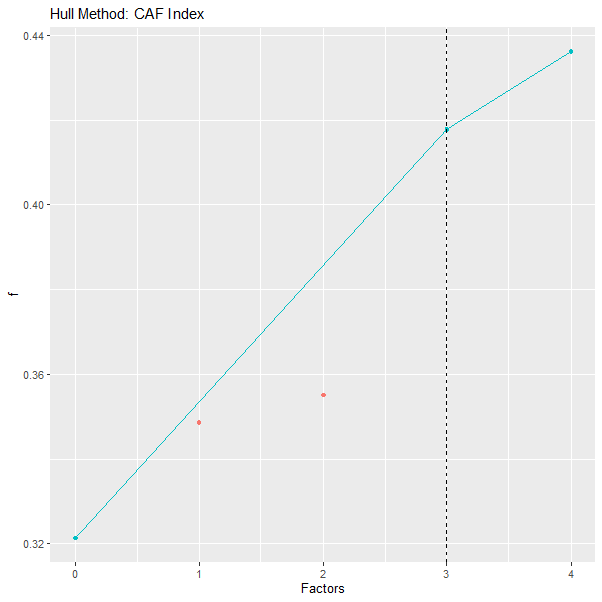

Supplement: S11 Fig — (TIF) [file pone.0238663.s011.tif]

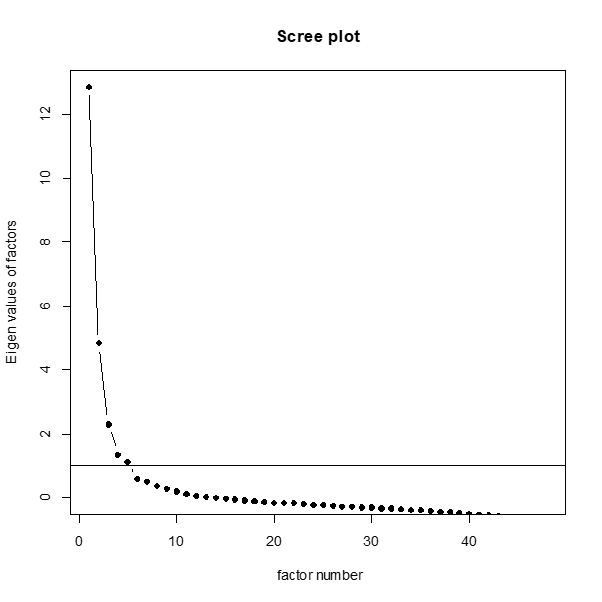

Supplement: S12 Fig — (TIF) [file pone.0238663.s012.tif]

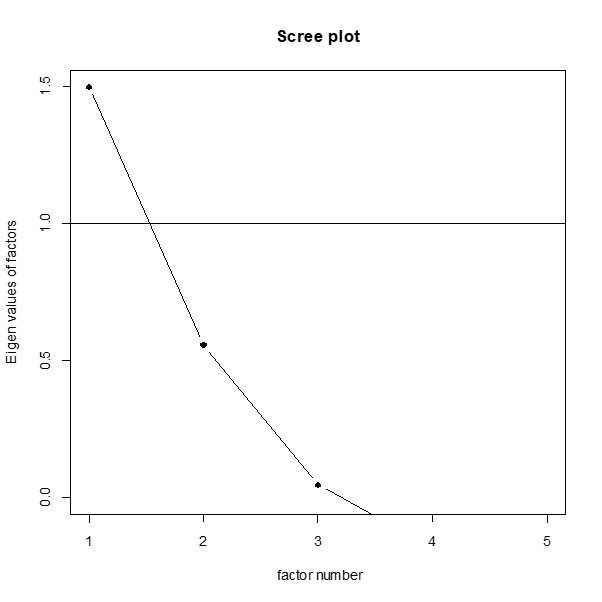

Supplement: S13 Fig — (TIF) [file pone.0238663.s013.tif]

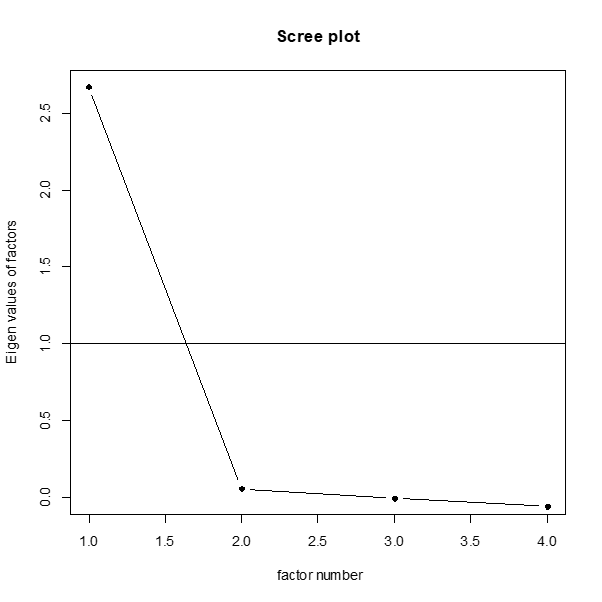

Supplement: S14 Fig — (TIF) [file pone.0238663.s014.tif]
