# Supplementary material for: Personality, subjective well-being, and the serotonin 1a receptor gene in common marmosets (Callithrix jacchus)
Source: PLoS One. 2021 Aug 9;16(8):e0238663. doi: 10.1371/journal.pone.0238663 (PMC8351977; doi:10.1371/journal.pone.0238663)
Supplement: S1 Table — a Substituted for “+ stingy”. b Substituted for “+ bold”. c Substituted for “+ cooperative”. d Substituted for “+ cool”. e Substituted for “− dependent”. (DOCX) [file pone.0238663.s015.docx]

Table S1

*Definitions of Factors from Previous Studies*

| Study / Factor | Definition |
| --- | --- |
| Iwanicki and Lehman (2014) |  |
| Extraversion | dominant + stingy/greedy^a^ − submissive + independent − timid^b^ + defiant + reckless − depressed − cautious − solitary |
| Agreeableness | affectionate + helpful^c^ + cool^d^ + aggressive − irritable + gentle |
| Conscientiousness | predictable + protective + conventional + intelligent |
| Openness | active + curious + inventive |
|  |  |
| Koski et al. (2017) |  |
| Conscientiousness | − thoughtless − bullying − clumsy − reckless − disorganized − imitative − erratic − jealous − aggressive − irritable − impulsive − excitable − depressed − stingy/greedy^a^ − playful |
| Agreeableness | friendly + affectionate + gentle + sociable + helpful + predictable + unemotional + protective |
| Assertiveness | − cautious − dependent/follower^e^ + dominant + independent − timid − submissive − fearful − vulnerable − sympathetic |
| Patience | − distractible + intelligent + inventive + sensitive |
| Inquisitive | − lazy + inquisitive + active − solitary |
|  |  |
| Inoue-Murayama et al. (2018) |  |
| Dominance | defiant + stingy/greedy + jealous + aggressive + dominant + irritable + bullying + excitable + impulsive − submissive − friendly − gentle − cool + disorganized + erratic + active + manipulative − conventional − predictable + distractable + thoughtless |
| Sociability | helpful − solitary + imitative + dependent/follower + protective − individualistic − independent + sociable + sympathetic + affectionate + playful + sensitive + curious + inquisitive − lazy |
| Neuroticism | timid − stable + autistic + fearful + vulnerable − intelligent + clumsy + depressed |

*Note*. ^a^ Substituted for “+ stingy”. ^b^ Substituted for “+ bold”. ^c^ Substituted for “+ cooperative”. ^d^ Substituted for “+ cool”. ^e^ Substituted for “− dependent”.
