# Supplementary material for: Personality, subjective well-being, and the serotonin 1a receptor gene in common marmosets (Callithrix jacchus)
Source: PLoS One. 2021 Aug 9;16(8):e0238663. doi: 10.1371/journal.pone.0238663 (PMC8351977; doi:10.1371/journal.pone.0238663)
Supplement: S2 Table — (DOCX) [file pone.0238663.s016.docx]

Table S2

*Congruence Coefficients for Comparison of Varimax- and Promax-Rotated Structures*

|  | ML1 | ML4 | ML5 | ML2 | ML3 |
| --- | --- | --- | --- | --- | --- |
| ML1 | 0.95 | -0.37 | -0.32 | 0.12 | -0.08 |
| ML4 | -0.35 | 0.92 | 0.39 | 0.22 | -0.18 |
| ML5 | -0.32 | 0.40 | 0.94 | 0.24 | -0.06 |
| ML2 | 0.12 | 0.14 | 0.14 | 0.97 | -0.23 |
| ML3 | -0.05 | -0.11 | -0.03 | -0.19 | 0.97 |
