# Supplementary material for: Personality, subjective well-being, and the serotonin 1a receptor gene in common marmosets (Callithrix jacchus)
Source: PLoS One. 2021 Aug 9;16(8):e0238663. doi: 10.1371/journal.pone.0238663 (PMC8351977; doi:10.1371/journal.pone.0238663)
Supplement: S3 Table — N = 128. Soc = Sociability, Dom = Dominance, Imp = Impulsiveness, Opn = Openness, Neg = Negative Affect, h2 = communalities. Factors extracted using a maximum likelihood estimation and rotated using the varimax procedure. Factor loadings greater than or equal to |0.4| are in bold. (DOCX) [file pone.0238663.s017.docx]

Table S3

*Pattern Matrix from the First-Order Factor Analysis of the Hominoid Personality Questionnaire*

|  | Factor Loadings | | | | |  |
| --- | --- | --- | --- | --- | --- | --- |
| Item | Soc | Dom | Imp | Opn | Neg | *h*^2^ |
| Helpful | **0.85** | -0.10 | -0.16 | 0.12 | -0.13 | 0.79 |
| Sympathetic | **0.82** | -0.21 | -0.20 | 0.06 | 0.04 | 0.76 |
| Protective | **0.77** | -0.14 | -0.11 | 0.06 | -0.17 | 0.66 |
| Individualistic | **-0.77** | 0.22 | 0.11 | 0.07 | 0.23 | 0.71 |
| Sociable | **0.74** | **-0.40** | -0.31 | 0.15 | -0.17 | 0.85 |
| Dependent/follower | **0.73** | -0.15 | -0.14 | 0.16 | 0.31 | 0.69 |
| Solitary | **-0.71** | 0.14 | 0.08 | -0.24 | 0.38 | 0.74 |
| Independent | **-0.71** | 0.32 | -0.08 | 0.06 | 0.05 | 0.62 |
| Affectionate | **0.69** | -0.27 | -0.27 | 0.17 | 0.07 | 0.65 |
| Sensitive | **0.67** | -0.26 | -0.29 | -0.05 | 0.03 | 0.61 |
| Imitative | **0.66** | -0.10 | -0.03 | 0.18 | 0.11 | 0.49 |
| Friendly | **0.66** | **-0.56** | -0.32 | 0.08 | 0.03 | 0.86 |
| Gentle | **0.65** | **-0.50** | -0.38 | 0.08 | 0.08 | 0.83 |
| Conventional | **0.61** | -0.15 | -0.39 | -0.18 | 0.19 | 0.62 |
| Intelligent | **0.55** | 0.07 | -0.20 | 0.04 | -0.22 | 0.39 |
| Reckless | **-0.50** | 0.10 | **0.43** | **0.40** | -0.08 | 0.62 |
| Jealous | -0.24 | **0.82** | 0.20 | 0.25 | 0.01 | 0.83 |
| Stingy/greedy | -0.31 | **0.79** | 0.20 | 0.30 | 0.01 | 0.86 |
| Bullying | -0.29 | **0.78** | 0.23 | 0.16 | -0.03 | 0.77 |
| Dominant | -0.34 | **0.76** | 0.27 | 0.09 | -0.15 | 0.80 |
| Aggressive | -0.36 | **0.71** | 0.33 | 0.05 | -0.22 | 0.80 |
| Defiant | -0.32 | **0.69** | 0.36 | 0.10 | -0.25 | 0.78 |
| Manipulative | 0.03 | **0.62** | 0.06 | 0.14 | **-0.40** | 0.57 |
| Irritable | -0.27 | **0.58** | **0.55** | -0.07 | -0.12 | 0.74 |
| Excitable | -0.26 | 0.37 | **0.75** | 0.03 | -0.07 | 0.78 |
| Impulsive | -0.30 | 0.28 | **0.74** | 0.20 | 0.10 | 0.76 |
| Unemotional | 0.04 | -0.09 | **-0.66** | -0.13 | 0.25 | 0.52 |
| Cool | 0.35 | -0.28 | **-0.66** | -0.13 | 0.02 | 0.65 |
| Disorganized | -0.27 | 0.30 | **0.54** | 0.22 | 0.07 | 0.51 |
| Distractible | -0.21 | 0.13 | **0.51** | 0.26 | 0.06 | 0.40 |
| Stable | **0.40** | -0.30 | **-0.50** | 0.07 | -0.36 | 0.64 |
| Fearful | 0.05 | -0.02 | **0.47** | **-0.40** | 0.36 | 0.51 |
| Thoughtless | -0.28 | 0.15 | **0.43** | **0.40** | -0.05 | 0.45 |
| Erratic | -0.39 | **0.41** | **0.43** | -0.03 | 0.18 | 0.54 |
| Predictable | 0.24 | -0.21 | **-0.41** | -0.02 | 0.02 | 0.27 |
| Curious | 0.13 | 0.15 | 0.14 | **0.73** | -0.14 | 0.61 |
| Inquisitive | 0.16 | 0.12 | 0.11 | **0.70** | -0.07 | 0.55 |
| Playful | 0.22 | -0.02 | 0.28 | **0.67** | -0.16 | 0.60 |
| Inventive | 0.26 | 0.12 | 0.00 | **0.65** | -0.05 | 0.51 |
| Active | 0.16 | 0.27 | **0.42** | **0.60** | -0.25 | 0.69 |
| Cautious | 0.31 | -0.02 | 0.12 | **-0.57** | 0.30 | 0.53 |
| Timid | 0.00 | -0.01 | 0.28 | -0.25 | **0.66** | 0.58 |
| Autistic | 0.02 | -0.16 | 0.06 | 0.06 | **0.64** | 0.45 |
| Depressed | -0.16 | -0.04 | -0.21 | -0.19 | **0.64** | 0.51 |
| Vulnerable | 0.01 | -0.23 | -0.06 | -0.08 | **0.57** | 0.39 |
| Clumsy | -0.11 | 0.09 | 0.00 | -0.10 | **0.55** | 0.33 |
| Lazy | -0.14 | -0.18 | **-0.40** | -0.31 | **0.53** | 0.59 |
| Submissive | **0.40** | -0.35 | -0.28 | -0.12 | **0.49** | 0.61 |
| Proportion variance | 0.20 | 0.13 | 0.12 | 0.08 | 0.08 |  |

*Note*. *N* = 128. Soc = Sociability, Dom = Dominance, Imp = Impulsiveness, Opn = Openness, Neg = Negative Affect, *h*^2^ = communalities. Factors extracted using a maximum likelihood estimation and rotated using the varimax procedure. Factor loadings greater than or equal to |0.4| are in bold.
