# Supplementary material for: Personality, subjective well-being, and the serotonin 1a receptor gene in common marmosets (Callithrix jacchus)
Source: PLoS One. 2021 Aug 9;16(8):e0238663. doi: 10.1371/journal.pone.0238663 (PMC8351977; doi:10.1371/journal.pone.0238663)
Supplement: S4 Table — N = 128. Factors were not assigned labels. h2 = communalities. Factors extracted using a maximum likelihood estimation and rotated using the promax procedure. Factor loadings greater than or equal to |0.4| are in bold. Correlation between factors = 0.07. (DOCX) [file pone.0238663.s018.docx]

Table S4

*Pattern Matrix from the Factor Analysis of Residualized Ratings*

|  | Factor | |  |
| --- | --- | --- | --- |
| Item | I | II | *h*^2^ |
| Friendly | **-0.90** | 0.11 | 0.81 |
| Gentle | **-0.89** | 0.09 | 0.80 |
| Sociable | **-0.86** | 0.36 | 0.84 |
| Sympathetic | **-0.80** | 0.24 | 0.67 |
| Aggressive | **0.79** | 0.22 | 0.69 |
| Dominant | **0.77** | 0.22 | 0.67 |
| Stingy/greedy | **0.76** | 0.28 | 0.69 |
| Bullying | **0.75** | 0.20 | 0.63 |
| Affectionate | **-0.75** | 0.20 | 0.58 |
| Defiant | **0.74** | 0.30 | 0.67 |
| Helpful | **-0.74** | **0.42** | 0.69 |
| Sensitive | **-0.74** | 0.12 | 0.55 |
| Irritable | **0.73** | 0.16 | 0.57 |
| Jealous | **0.72** | 0.28 | 0.62 |
| Individualistic | **0.72** | -0.31 | 0.58 |
| Excitable | **0.70** | 0.19 | 0.54 |
| Protective | **-0.69** | 0.38 | 0.59 |
| Cool | **-0.68** | -0.16 | 0.50 |
| Conventional | **-0.68** | -0.12 | 0.48 |
| Erratic | **0.68** | -0.09 | 0.46 |
| Impulsive | **0.67** | 0.18 | 0.50 |
| Stable | **-0.67** | 0.24 | 0.48 |
| Independent | **0.66** | -0.19 | 0.46 |
| Disorganized | **0.61** | 0.19 | 0.43 |
| Dependent/follower | **-0.61** | 0.21 | 0.40 |
| Reckless | **0.58** | 0.25 | 0.42 |
| Submissive | **-0.57** | -0.35 | 0.48 |
| Intelligent | **-0.50** | 0.29 | 0.31 |
| Imitative | **-0.49** | 0.31 | 0.32 |
| Predictable | **-0.47** | -0.08 | 0.23 |
| Thoughtless | **0.45** | 0.29 | 0.30 |
| Distractible | **0.45** | 0.15 | 0.23 |
| Active | 0.23 | **0.74** | 0.63 |
| Lazy | -0.09 | **-0.70** | 0.51 |
| Curious | 0.07 | **0.64** | 0.42 |
| Playful | -0.05 | **0.64** | 0.40 |
| Solitary | **0.60** | **-0.62** | 0.69 |
| Inquisitive | 0.06 | **0.61** | 0.38 |
| Inventive | -0.05 | **0.61** | 0.37 |
| Depressed | 0.03 | **-0.59** | 0.35 |
| Timid | 0.15 | **-0.47** | 0.24 |
| Manipulative | 0.35 | **0.46** | 0.36 |
| Vulnerable | -0.11 | **-0.44** | 0.21 |
| Clumsy | 0.16 | -0.38 | 0.17 |
| Cautious | -0.15 | -0.37 | 0.17 |
| Unemotional | -0.29 | -0.37 | 0.23 |
| Autistic | 0.02 | -0.35 | 0.12 |
| Fearful | 0.17 | -0.32 | 0.12 |
| Proportion of variance | 0.34 | 0.13 |  |

*Note*. *N* = 128. Factors were not assigned labels. *h*^2^ = communalities. Factors extracted using a maximum likelihood estimation and rotated using the promax procedure. Factor loadings greater than or equal to |0.4| are in bold. Correlation between factors = 0.07.
