# Supplementary material for: Personality, subjective well-being, and the serotonin 1a receptor gene in common marmosets (Callithrix jacchus)
Source: PLoS One. 2021 Aug 9;16(8):e0238663. doi: 10.1371/journal.pone.0238663 (PMC8351977; doi:10.1371/journal.pone.0238663)
Supplement: S5 Table — N = 128. Factors were not assigned labels. h2 = communalities. Factors extracted using a maximum likelihood estimation and rotated using the promax procedure. Factor loadings greater than or equal to |0.4| are in bold. (DOCX) [file pone.0238663.s019.docx]

Table S5

*Pattern Matrix from the Factor Analysis of Residualized Ratings*

|  | Factor | | | |  |
| --- | --- | --- | --- | --- | --- |
| Item | I | II | III | IV | *h*^2^ |
| Independent | **-0.83** | -0.10 | -0.06 | 0.07 | 0.63 |
| Helpful | **0.81** | -0.05 | -0.19 | 0.04 | 0.75 |
| Individualistic | **-0.80** | 0.02 | 0.22 | 0.12 | 0.69 |
| Dependent/follower | **0.78** | 0.09 | 0.23 | 0.14 | 0.61 |
| Sympathetic | **0.78** | -0.12 | 0.00 | 0.02 | 0.73 |
| Protective | **0.75** | -0.04 | -0.20 | 0.01 | 0.64 |
| Imitative | **0.71** | 0.15 | 0.07 | 0.13 | 0.45 |
| Solitary | **-0.70** | 0.04 | 0.34 | -0.19 | 0.71 |
| Sociable | **0.67** | -0.34 | -0.13 | 0.17 | 0.83 |
| Sensitive | **0.63** | -0.20 | -0.05 | -0.09 | 0.58 |
| Friendly | **0.59** | **-0.45** | 0.14 | 0.15 | 0.84 |
| Affectionate | **0.58** | -0.29 | 0.08 | 0.18 | 0.60 |
| Gentle | **0.55** | **-0.48** | 0.14 | 0.15 | 0.82 |
| Conventional | **0.49** | -0.24 | 0.02 | -0.26 | 0.52 |
| Intelligent | **0.42** | -0.17 | -0.34 | -0.06 | 0.37 |
| Excitable | 0.04 | **0.86** | 0.02 | 0.00 | 0.71 |
| Irritable | -0.07 | **0.80** | -0.19 | -0.18 | 0.71 |
| Impulsive | 0.00 | **0.79** | 0.22 | 0.20 | 0.72 |
| Cool | 0.09 | **-0.69** | -0.09 | -0.13 | 0.60 |
| Fearful | 0.32 | **0.63** | 0.39 | -0.38 | 0.51 |
| Stable | 0.20 | **-0.62** | -0.37 | 0.07 | 0.64 |
| Disorganized | -0.09 | **0.61** | 0.13 | 0.22 | 0.52 |
| Unemotional | -0.27 | **-0.59** | 0.02 | -0.16 | 0.34 |
| Erratic | -0.22 | **0.59** | 0.13 | -0.08 | 0.51 |
| Defiant | -0.25 | **0.58** | **-0.41** | -0.05 | 0.75 |
| Jealous | -0.24 | **0.56** | -0.27 | 0.04 | 0.64 |
| Aggressive | -0.33 | **0.56** | -0.38 | -0.09 | 0.76 |
| Dominant | -0.32 | **0.55** | -0.36 | -0.08 | 0.73 |
| Bullying | -0.31 | **0.54** | -0.27 | -0.02 | 0.65 |
| Stingy/greedy | -0.33 | **0.51** | -0.24 | 0.11 | 0.69 |
| Predictable | 0.06 | **-0.49** | -0.02 | 0.00 | 0.27 |
| Distractible | -0.08 | **0.42** | 0.23 | 0.30 | 0.35 |
| Autistic | -0.02 | 0.04 | **0.68** | 0.15 | 0.42 |
| Timid | 0.14 | **0.41** | **0.64** | -0.22 | 0.58 |
| Manipulative | -0.05 | 0.32 | **-0.63** | -0.05 | 0.53 |
| Vulnerable | -0.01 | -0.09 | **0.61** | 0.02 | 0.39 |
| Depressed | -0.25 | -0.16 | **0.51** | -0.14 | 0.40 |
| Submissive | 0.32 | -0.28 | **0.48** | -0.06 | 0.58 |
| Clumsy | -0.11 | 0.12 | **0.43** | -0.09 | 0.24 |
| Lazy | -0.33 | -0.37 | **0.42** | -0.26 | 0.52 |
| Playful | 0.26 | 0.11 | 0.04 | **0.72** | 0.60 |
| Curious | 0.09 | 0.06 | -0.06 | **0.71** | 0.57 |
| Inquisitive | 0.12 | 0.09 | -0.04 | **0.67** | 0.52 |
| Cautious | **0.45** | **0.40** | 0.15 | **-0.66** | 0.54 |
| Inventive | 0.19 | 0.05 | -0.09 | **0.60** | 0.45 |
| Reckless | -0.36 | 0.24 | 0.17 | **0.56** | 0.61 |
| Active | 0.27 | **0.45** | -0.17 | **0.54** | 0.68 |
| Thoughtless | -0.16 | 0.31 | 0.15 | **0.47** | 0.43 |
| Proportion of variance | 0.20 | 0.20 | 0.09 | 0.09 |  |
|  |  |  |  |  |  |
|  | Factor Correlations | | | |  |
|  | I | II | III | IV |  |
| I | 1.00 |  |  |  |  |
| II | -0.52 | 1.00 |  |  |  |
| III | 0.00 | -0.11 | 1.00 |  |  |
| IV | 0.02 | 0.25 | -0.33 | 1.00 |  |

*Note*. *N* = 128. Factors were not assigned labels. *h*^2^ = communalities. Factors extracted using a maximum likelihood estimation and rotated using the promax procedure. Factor loadings greater than or equal to |0.4| are in bold.
