# Supplementary material for: Personality, subjective well-being, and the serotonin 1a receptor gene in common marmosets (Callithrix jacchus)
Source: PLoS One. 2021 Aug 9;16(8):e0238663. doi: 10.1371/journal.pone.0238663 (PMC8351977; doi:10.1371/journal.pone.0238663)
Supplement: S6 Table — N = 128. Soc = Sociability, Dom = Dominance, Imp = Impulsiveness, Opn = Openness, Neg = Negative Affect, h2 = communalities. Factors extracted using a maximum likelihood estimation and rotated using the promax procedure. Factor loadings greater than or equal to |0.4| are in bold. (DOCX) [file pone.0238663.s020.docx]

Table S6

*Pattern Matrix from the Factor Analysis of Residualized Ratings*

|  | Factor | | | | |  |
| --- | --- | --- | --- | --- | --- | --- |
| Item | Soc | Dom | Imp | Opn | Neg | *h*^2^ |
| Dependent/follower | **0.85** | 0.02 | 0.07 | 0.21 | 0.33 | 0.67 |
| Helpful | **0.85** | 0.07 | -0.09 | 0.10 | -0.08 | 0.77 |
| Sympathetic | **0.84** | 0.00 | -0.13 | 0.09 | 0.11 | 0.77 |
| Protective | **0.78** | 0.08 | -0.09 | 0.06 | -0.10 | 0.66 |
| Individualistic | **-0.70** | 0.13 | -0.08 | 0.15 | 0.29 | 0.70 |
| Imitative | **0.69** | -0.04 | 0.18 | 0.14 | 0.07 | 0.45 |
| Independent | **-0.68** | 0.31 | -0.31 | 0.14 | 0.12 | 0.66 |
| Sensitive | **0.64** | -0.08 | -0.16 | -0.05 | 0.00 | 0.59 |
| Affectionate | **0.63** | -0.10 | -0.24 | 0.25 | 0.18 | 0.65 |
| Conventional | **0.59** | 0.07 | -0.31 | -0.17 | 0.18 | 0.59 |
| Solitary | **-0.57** | 0.19 | -0.13 | -0.13 | **0.46** | 0.75 |
| Sociable | **0.57** | -0.35 | -0.10 | 0.13 | -0.22 | 0.84 |
| Intelligent | **0.54** | 0.27 | -0.35 | 0.04 | -0.12 | 0.43 |
| Gentle | **0.53** | -0.34 | -0.26 | 0.18 | 0.13 | 0.83 |
| Jealous | 0.02 | **0.84** | 0.02 | 0.16 | 0.07 | 0.77 |
| Dominant | -0.11 | **0.79** | 0.04 | 0.01 | -0.08 | 0.79 |
| Stingy/greedy | -0.09 | **0.77** | 0.01 | 0.24 | 0.08 | 0.80 |
| Bullying | -0.09 | **0.75** | 0.05 | 0.08 | 0.01 | 0.72 |
| Aggressive | -0.16 | **0.73** | 0.09 | -0.02 | -0.14 | 0.79 |
| Defiant | -0.08 | **0.73** | 0.12 | 0.02 | -0.17 | 0.77 |
| Manipulative | 0.12 | **0.68** | -0.11 | 0.04 | -0.36 | 0.56 |
| Irritable | 0.04 | **0.64** | 0.38 | -0.14 | -0.05 | 0.71 |
| Friendly | **0.45** | **-0.56** | -0.07 | 0.09 | -0.04 | 0.85 |
| Erratic | -0.09 | **0.47** | 0.27 | -0.03 | 0.26 | 0.54 |
| Impulsive | -0.03 | 0.18 | **0.68** | 0.14 | 0.13 | 0.72 |
| Cool | 0.19 | -0.06 | **-0.68** | -0.02 | 0.08 | 0.66 |
| Fearful | 0.21 | -0.07 | **0.66** | **-0.48** | 0.19 | 0.55 |
| Unemotional | -0.13 | 0.04 | **-0.66** | -0.05 | 0.23 | 0.43 |
| Excitable | 0.03 | 0.34 | **0.65** | -0.05 | -0.01 | 0.71 |
| Stable | 0.18 | -0.19 | **-0.48** | 0.09 | -0.34 | 0.64 |
| Distractible | -0.14 | -0.05 | **0.47** | 0.24 | 0.10 | 0.37 |
| Disorganized | -0.07 | 0.23 | **0.46** | 0.20 | 0.12 | 0.52 |
| Predictable | 0.12 | -0.09 | **-0.44** | 0.07 | 0.08 | 0.29 |
| Curious | 0.17 | 0.13 | -0.01 | **0.77** | 0.06 | 0.61 |
| Inquisitive | 0.17 | 0.08 | 0.07 | **0.70** | 0.03 | 0.53 |
| Playful | 0.23 | -0.12 | 0.22 | **0.69** | 0.00 | 0.59 |
| Inventive | 0.30 | 0.19 | -0.06 | **0.68** | 0.07 | 0.51 |
| Cautious | **0.44** | 0.14 | 0.27 | **-0.65** | 0.14 | 0.54 |
| Active | 0.25 | 0.16 | 0.39 | **0.51** | -0.18 | 0.68 |
| Reckless | **-0.41** | -0.09 | 0.32 | **0.50** | 0.06 | 0.62 |
| Thoughtless | -0.19 | -0.02 | 0.35 | **0.42** | 0.06 | 0.44 |
| Depressed | -0.10 | 0.06 | -0.25 | -0.03 | **0.68** | 0.52 |
| Autistic | 0.02 | -0.17 | 0.12 | 0.18 | **0.67** | 0.45 |
| Timid | 0.15 | -0.02 | 0.39 | -0.22 | **0.59** | 0.58 |
| Clumsy | 0.03 | 0.18 | -0.03 | 0.00 | **0.58** | 0.33 |
| Vulnerable | 0.01 | -0.23 | 0.02 | 0.04 | **0.58** | 0.40 |
| Submissive | 0.37 | -0.21 | -0.18 | 0.01 | **0.54** | 0.63 |
| Lazy | -0.24 | -0.09 | -0.36 | -0.18 | **0.51** | 0.57 |
| Proportion of variance | 0.18 | 0.15 | 0.12 | 0.09 | 0.08 |  |
|  |  |  |  |  |  |  |
|  | Factor Correlations | | | | |  |
|  | Soc | Dom | Imp | Opn | Neg |  |
| Soc | 1.00 |  |  |  |  |  |
| Dom | -0.56 | 1.00 |  |  |  |  |
| Imp | -0.40 | 0.50 | 1.00 |  |  |  |
| Opn | -0.02 | 0.21 | 0.23 | 1.00 |  |  |
| Neg | -0.17 | -0.12 | 0.07 | -0.38 | 1.00 |  |

*Note*. *N* = 128. Soc = Sociability, Dom = Dominance, Imp = Impulsiveness, Opn = Openness, Neg = Negative Affect, *h*^2^ = communalities. Factors extracted using a maximum likelihood estimation and rotated using the promax procedure. Factor loadings greater than or equal to |0.4| are in bold.
