# Supplementary material for: Personality, subjective well-being, and the serotonin 1a receptor gene in common marmosets (Callithrix jacchus)
Source: PLoS One. 2021 Aug 9;16(8):e0238663. doi: 10.1371/journal.pone.0238663 (PMC8351977; doi:10.1371/journal.pone.0238663)
Supplement: S7 Table — (DOCX) [file pone.0238663.s021.docx]

Table S7

*Congruence Coefficients for Comparison of Factors from Raw and Residualized Scores*

|  | MR5 | MR1 | MR4 | MR2 | MR3 |
| --- | --- | --- | --- | --- | --- |
| ML1 | 0.99 | -0.16 | -0.11 | 0.09 | -0.08 |
| ML4 | -0.12 | 0.98 | 0.12 | 0.10 | -0.07 |
| ML5 | -0.09 | 0.23 | 0.98 | 0.11 | -0.04 |
| ML2 | 0.15 | 0.07 | 0.06 | 0.99 | -0.02 |
| ML3 | 0.03 | -0.06 | 0.04 | -0.04 | 0.98 |
