# Supplementary material for: Personality, subjective well-being, and the serotonin 1a receptor gene in common marmosets (Callithrix jacchus)
Source: PLoS One. 2021 Aug 9;16(8):e0238663. doi: 10.1371/journal.pone.0238663 (PMC8351977; doi:10.1371/journal.pone.0238663)
Supplement: S8 Table — N = 128. Imp = Impulsiveness, Soc = Sociability, Dom = Dominance, Opn = Openness, Neg = Negative Affect, h2 = communalities. Factors extracted using a maximum likelihood estimation and rotated using the promax procedure. Factor loadings greater than or equal to |0.4| are in bold. (DOCX) [file pone.0238663.s022.docx]

Table S8

*Pattern Matrix from the Factor Analysis of Rater 1’s Ratings*

|  | Factor | | | | |  |
| --- | --- | --- | --- | --- | --- | --- |
| Item | Imp | Soc | Dom | Opn | Neg | *h*^2^ |
| Impulsive | **0.94** | 0.06 | -0.07 | -0.01 | 0.05 | 0.77 |
| Excitable | **0.91** | 0.02 | -0.10 | -0.03 | 0.01 | 0.74 |
| Cool | **-0.74** | 0.01 | -0.08 | 0.04 | 0.03 | 0.63 |
| Erratic | **0.74** | 0.05 | 0.15 | -0.08 | -0.01 | 0.65 |
| Distractible | **0.69** | -0.21 | -0.07 | 0.20 | 0.05 | 0.62 |
| Stable | **-0.68** | -0.11 | -0.26 | 0.05 | -0.08 | 0.61 |
| Unemotional | **-0.67** | 0.10 | -0.02 | -0.06 | 0.27 | 0.67 |
| Disorganized | **0.65** | -0.19 | -0.01 | 0.09 | 0.07 | 0.58 |
| Predictable | **-0.64** | 0.21 | 0.15 | -0.28 | 0.03 | 0.53 |
| Reckless | **0.64** | -0.33 | -0.03 | 0.22 | 0.03 | 0.72 |
| Fearful | **0.60** | 0.26 | 0.02 | -0.30 | 0.25 | 0.47 |
| Irritable | **0.58** | 0.14 | 0.36 | -0.18 | -0.26 | 0.69 |
| Thoughtless | **0.57** | -0.19 | 0.02 | 0.28 | 0.06 | 0.50 |
| Timid | **0.54** | 0.08 | 0.16 | -0.06 | **0.42** | 0.51 |
| Clumsy | 0.32 | -0.02 | -0.11 | -0.09 | 0.30 | 0.20 |
| Protective | 0.20 | **0.87** | -0.13 | 0.07 | -0.35 | 0.77 |
| Helpful | 0.00 | **0.85** | -0.03 | 0.11 | -0.24 | 0.81 |
| Intelligent | -0.12 | **0.78** | 0.15 | -0.09 | -0.12 | 0.58 |
| Sympathetic | -0.03 | **0.76** | -0.15 | 0.03 | -0.17 | 0.75 |
| Affectionate | 0.00 | **0.63** | -0.19 | 0.18 | -0.12 | 0.65 |
| Sensitive | -0.24 | **0.61** | 0.04 | 0.13 | 0.24 | 0.72 |
| Dependent/follower | -0.02 | **0.57** | -0.07 | 0.39 | 0.20 | 0.65 |
| Independent | 0.13 | **-0.54** | 0.13 | 0.07 | -0.01 | 0.50 |
| Individualistic | 0.24 | **-0.48** | 0.23 | 0.15 | 0.18 | 0.61 |
| Imitative | -0.15 | **0.46** | 0.11 | 0.33 | 0.25 | 0.46 |
| Gentle | -0.28 | **0.45** | -0.29 | 0.13 | 0.08 | 0.83 |
| Cautious | 0.29 | **0.45** | -0.03 | -0.34 | 0.28 | 0.40 |
| Solitary | 0.21 | **-0.42** | 0.16 | -0.22 | 0.26 | 0.61 |
| Conventional | -0.38 | **0.41** | 0.03 | -0.10 | 0.36 | 0.67 |
| Sociable | -0.25 | **0.40** | -0.38 | 0.19 | -0.06 | 0.82 |
| Bullying | 0.02 | 0.01 | **0.91** | 0.05 | -0.02 | 0.84 |
| Aggressive | 0.03 | -0.05 | **0.86** | -0.01 | -0.11 | 0.88 |
| Jealous | -0.03 | -0.07 | **0.86** | 0.21 | 0.13 | 0.76 |
| Dominant | -0.01 | -0.05 | **0.85** | 0.03 | -0.09 | 0.80 |
| Stingy/greedy | 0.04 | -0.21 | **0.78** | 0.23 | 0.20 | 0.82 |
| Defiant | 0.07 | 0.02 | **0.78** | 0.02 | -0.13 | 0.70 |
| Manipulative | -0.17 | **0.47** | **0.51** | 0.23 | -0.21 | 0.42 |
| Friendly | -0.30 | 0.23 | **-0.42** | 0.25 | 0.22 | 0.83 |
| Curious | 0.09 | 0.10 | 0.07 | **0.80** | 0.07 | 0.61 |
| Inquisitive | -0.01 | 0.11 | 0.03 | **0.78** | 0.02 | 0.64 |
| Inventive | -0.04 | 0.14 | 0.12 | **0.76** | 0.15 | 0.57 |
| Playful | 0.16 | 0.12 | -0.05 | **0.76** | 0.01 | 0.58 |
| Active | 0.36 | 0.01 | 0.14 | **0.69** | -0.03 | 0.59 |
| Autistic | 0.06 | -0.09 | -0.07 | 0.39 | **0.66** | 0.41 |
| Vulnerable | 0.03 | -0.19 | -0.04 | 0.01 | **0.51** | 0.27 |
| Submissive | -0.14 | 0.31 | -0.14 | 0.06 | **0.47** | 0.54 |
| Lazy | -0.30 | -0.06 | -0.02 | -0.19 | **0.43** | 0.34 |
| Depressed | -0.18 | -0.21 | 0.27 | -0.12 | **0.43** | 0.29 |
| Proportion of variance | 0.19 | 0.16 | 0.13 | 0.09 | 0.05 |  |
|  |  |  |  |  |  |  |
|  | Factor Correlations | | | | |  |
|  | Imp | Soc | Dom | Opn | Neg |  |
| Imp | 1.00 |  |  |  |  |  |
| Soc | -0.62 | 1.00 |  |  |  |  |
| Dom | 0.51 | -0.55 | 1.00 |  |  |  |
| Opn | -0.15 | 0.18 | -0.08 | 1.00 |  |  |
| Neg | -0.04 | 0.15 | -0.17 | -0.34 | 1.00 |  |

*Note*. *N* = 128. Imp = Impulsiveness, Soc = Sociability, Dom = Dominance, Opn = Openness, Neg = Negative Affect, *h*^2^ = communalities. Factors extracted using a maximum likelihood estimation and rotated using the promax procedure. Factor loadings greater than or equal to |0.4| are in bold.
