# Supplementary material for: Personality, subjective well-being, and the serotonin 1a receptor gene in common marmosets (Callithrix jacchus)
Source: PLoS One. 2021 Aug 9;16(8):e0238663. doi: 10.1371/journal.pone.0238663 (PMC8351977; doi:10.1371/journal.pone.0238663)
Supplement: S9 Table — N = 128. I = Pro-sociality (reversed), II = Boldness, h2 = communalities. 1 When rounded to three decimal places, this loading was equal to 0.995. Factors extracted using a maximum likelihood estimation and rotated using the promax procedure. Factor loadings greater than or equal to |0.4| are in bold. Factor correlation = -0.14. (DOCX) [file pone.0238663.s023.docx]

Table S9

*Pattern Matrix from Second-Order Factor Analysis from Rater 1’s Ratings*

|  | Second-order Factor | |  |
| --- | --- | --- | --- |
| First-order Factor | I | II | *h*^2^ |
| Sociability | **-0.83** | 0.03 | 0.68 |
| Impulsivity | **0.74** | 0.07 | 0.57 |
| Dominance | **0.68** | -0.07 | 0.45 |
| Negative affect | -0.29 | **< 1.00** | < 1.00 |
| Openness | -0.14 | -0.38 | 0.18 |
| Proportion of variance | 0.35 | 0.22 |  |

*Note*. *N* = 128. I = Pro-sociality (reversed), II = Boldness, *h*^2^ = communalities. ^1^ When rounded to three decimal places, this loading was equal to 0.995. Factors extracted using a maximum likelihood estimation and rotated using the promax procedure. Factor loadings greater than or equal to |0.4| are in bold. Factor correlation = -0.14.
