# Supplementary material for: Personality, subjective well-being, and the serotonin 1a receptor gene in common marmosets (Callithrix jacchus)
Source: PLoS One. 2021 Aug 9;16(8):e0238663. doi: 10.1371/journal.pone.0238663 (PMC8351977; doi:10.1371/journal.pone.0238663)
Supplement: S10 Table — N = 128. Factors were not assigned labels. h2 = communalities. Factors extracted using a maximum likelihood estimation and rotated using the promax procedure. Factor loadings greater than or equal to |0.4| are in bold. (DOCX) [file pone.0238663.s024.docx]

Table S10

*Pattern Matrix from the Factor Analysis of Rater 2’s Ratings*

|  | Factor | | | |  |
| --- | --- | --- | --- | --- | --- |
| Item | I | II | III | IV | *h*^2^ |
| Aggressive | **0.81** | 0.30 | -0.19 | -0.16 | 0.64 |
| Irritable | **0.80** | -0.01 | 0.10 | -0.08 | 0.59 |
| Excitable | **0.79** | 0.17 | 0.08 | -0.09 | 0.56 |
| Dominant | **0.79** | 0.33 | -0.28 | -0.13 | 0.67 |
| Defiant | **0.75** | 0.24 | -0.09 | -0.16 | 0.51 |
| Gentle | **-0.73** | 0.31 | 0.07 | 0.12 | 0.71 |
| Manipulative | **0.72** | **0.40** | -0.13 | -0.10 | 0.54 |
| Submissive | **-0.70** | -0.13 | 0.03 | -0.02 | 0.49 |
| Affectionate | **-0.64** | 0.33 | 0.13 | 0.07 | 0.66 |
| Friendly | **-0.58** | 0.20 | 0.38 | 0.11 | 0.70 |
| Vulnerable | **-0.54** | -0.10 | -0.08 | -0.10 | 0.34 |
| Bullying | **0.53** | 0.01 | -0.17 | 0.03 | 0.36 |
| Sympathetic | **-0.52** | 0.18 | 0.35 | -0.04 | 0.59 |
| Stingy/greedy | **0.51** | -0.04 | -0.20 | 0.16 | 0.43 |
| Jealous | **0.48** | 0.01 | -0.16 | 0.08 | 0.31 |
| Lazy | **-0.48** | **-0.43** | -0.28 | -0.21 | 0.62 |
| Impulsive | **0.48** | -0.29 | 0.16 | 0.27 | 0.47 |
| Depressed | **-0.46** | -0.39 | -0.24 | -0.13 | 0.47 |
| Cool | **-0.45** | 0.39 | -0.21 | -0.30 | 0.52 |
| Helpful | **-0.40** | 0.30 | 0.38 | -0.03 | 0.59 |
| Predictable | -0.31 | 0.02 | -0.13 | 0.30 | 0.11 |
| Conventional | -0.31 | 0.25 | 0.13 | -0.25 | 0.33 |
| Intelligent | 0.20 | **0.73** | -0.07 | -0.01 | 0.49 |
| Timid | -0.38 | **-0.64** | 0.08 | -0.21 | 0.58 |
| Sensitive | -0.16 | **0.62** | 0.04 | -0.19 | 0.49 |
| Stable | -0.10 | **0.57** | 0.15 | -0.07 | 0.45 |
| Clumsy | -0.25 | **-0.54** | -0.08 | 0.07 | 0.32 |
| Sociable | -0.34 | **0.43** | 0.37 | 0.09 | 0.68 |
| Protective | -0.37 | **0.42** | 0.33 | 0.01 | 0.63 |
| Inventive | 0.05 | **0.41** | -0.01 | 0.16 | 0.21 |
| Erratic | 0.26 | -0.34 | 0.04 | -0.09 | 0.21 |
| Autistic | -0.15 | -0.30 | -0.06 | -0.02 | 0.12 |
| Disorganized | 0.20 | -0.27 | 0.18 | 0.19 | 0.16 |
| Independent | 0.07 | 0.10 | **-0.85** | 0.08 | 0.69 |
| Individualistic | 0.06 | -0.03 | **-0.84** | 0.08 | 0.75 |
| Imitative | -0.15 | -0.05 | **0.70** | -0.07 | 0.53 |
| Solitary | 0.05 | -0.28 | **-0.62** | -0.05 | 0.63 |
| Dependent/follower | **-0.42** | -0.12 | **0.49** | -0.12 | 0.48 |
| Unemotional | 0.02 | -0.06 | -0.31 | -0.11 | 0.14 |
| Reckless | -0.06 | -0.06 | -0.07 | **0.83** | 0.63 |
| Thoughtless | -0.01 | -0.01 | 0.00 | **0.74** | 0.54 |
| Curious | 0.10 | 0.29 | -0.06 | **0.72** | 0.70 |
| Playful | -0.02 | 0.20 | 0.17 | **0.66** | 0.60 |
| Cautious | 0.29 | 0.01 | 0.15 | **-0.65** | 0.35 |
| Inquisitive | 0.09 | 0.05 | 0.06 | **0.46** | 0.27 |
| Fearful | 0.13 | **-0.42** | 0.26 | **-0.44** | 0.35 |
| Active | 0.37 | 0.26 | 0.31 | **0.40** | 0.61 |
| Distractible | 0.04 | -0.07 | 0.04 | 0.38 | 0.15 |
| Proportion of variance | 0.19 | 0.10 | 0.10 | 0.09 |  |
|  |  |  |  |  |  |
|  | Factor Correlations | | | |  |
|  | I | II | III | IV |  |
| I | 1.00 |  |  |  |  |
| II | -0.17 | 1.00 |  |  |  |
| III | -0.19 | 0.41 | 1.00 |  |  |
| IV | 0.36 | 0.16 | 0.14 | 1.00 |  |

*Note*. *N* = 128. Factors were not assigned labels. *h*^2^ = communalities. Factors extracted using a maximum likelihood estimation and rotated using the promax procedure. Factor loadings greater than or equal to |0.4| are in bold.
