# Supplementary material for: Personality, subjective well-being, and the serotonin 1a receptor gene in common marmosets (Callithrix jacchus)
Source: PLoS One. 2021 Aug 9;16(8):e0238663. doi: 10.1371/journal.pone.0238663 (PMC8351977; doi:10.1371/journal.pone.0238663)
Supplement: S11 Table — N = 81. Factors were not assigned labels. h2 = communalities. Factors extracted using a maximum likelihood estimation and rotated using the promax procedure. Factor loadings greater than or equal to |0.4| are in bold. (DOCX) [file pone.0238663.s025.docx]

Table S11

*Pattern Matrix from the Factor Analysis of Rater 3’s Ratings*

|  | Factor | | |  |
| --- | --- | --- | --- | --- |
| Item | I | II | III | *h*^2^ |
| Aggressive | **-0.89** | -0.08 | -0.05 | 0.761 |
| Gentle | **0.82** | 0.15 | -0.03 | 0.687 |
| Dominant | **-0.82** | 0.02 | 0.04 | 0.683 |
| Bullying | **-0.80** | 0.03 | 0.00 | 0.649 |
| Defiant | **-0.80** | 0.06 | -0.02 | 0.646 |
| Stingy/greedy | **-0.80** | 0.11 | -0.05 | 0.648 |
| Friendly | **0.78** | 0.19 | 0.08 | 0.596 |
| Affectionate | **0.72** | 0.19 | 0.07 | 0.509 |
| Irritable | **-0.71** | -0.05 | 0.20 | 0.605 |
| Submissive | **0.68** | -0.11 | 0.25 | 0.474 |
| Jealous | **-0.66** | 0.12 | 0.17 | 0.537 |
| Cautious | **0.58** | -0.19 | 0.01 | 0.388 |
| Sociable | **0.58** | 0.39 | -0.18 | 0.535 |
| Intelligent | **0.55** | 0.07 | 0.00 | 0.305 |
| Sympathetic | **0.55** | **0.49** | 0.14 | 0.475 |
| Reckless | **-0.52** | 0.02 | 0.17 | 0.341 |
| Excitable | **-0.52** | -0.01 | **0.48** | 0.613 |
| Manipulative | **-0.49** | 0.16 | -0.18 | 0.270 |
| Cool | **0.47** | -0.19 | **-0.46** | 0.579 |
| Protective | **0.47** | 0.36 | 0.00 | 0.319 |
| Autistic | **0.46** | -0.18 | 0.34 | 0.299 |
| Sensitive | **0.46** | 0.19 | 0.00 | 0.232 |
| Erratic | **-0.45** | -0.04 | 0.14 | 0.252 |
| Disorganized | **-0.43** | 0.09 | 0.08 | 0.223 |
| Conventional | **0.40** | 0.03 | -0.24 | 0.267 |
| Thoughtless | -0.39 | 0.25 | 0.20 | 0.309 |
| Solitary | -0.16 | **-0.70** | 0.14 | 0.525 |
| Active | -0.32 | **0.59** | 0.09 | 0.506 |
| Depressed | 0.24 | **-0.58** | 0.33 | 0.497 |
| Lazy | 0.21 | **-0.54** | -0.18 | 0.405 |
| Helpful | **0.46** | **0.52** | 0.04 | 0.433 |
| Imitative | 0.17 | **0.51** | 0.04 | 0.276 |
| Playful | 0.15 | **0.50** | 0.07 | 0.257 |
| Inquisitive | -0.15 | **0.45** | -0.15 | 0.251 |
| Independent | -0.33 | **-0.41** | -0.16 | 0.254 |
| Curious | -0.07 | 0.35 | 0.13 | 0.149 |
| Clumsy | 0.14 | -0.30 | 0.27 | 0.174 |
| Dependent/follower | 0.10 | 0.24 | 0.02 | 0.063 |
| Inventive | -0.10 | 0.18 | -0.01 | 0.044 |
| Timid | 0.20 | -0.12 | **0.72** | 0.506 |
| Stable | 0.06 | 0.04 | **-0.64** | 0.434 |
| Impulsive | -0.19 | 0.15 | **0.62** | 0.496 |
| Unemotional | 0.32 | -0.25 | **-0.61** | 0.642 |
| Vulnerable | 0.35 | -0.22 | **0.57** | 0.422 |
| Predictable | 0.30 | -0.08 | **-0.47** | 0.390 |
| Fearful | 0.05 | 0.00 | **0.42** | 0.169 |
| Distractible | -0.08 | 0.23 | 0.39 | 0.225 |
| Individualistic | -0.22 | -0.14 | 0.30 | 0.190 |
| Proportion of variance | 0.24 | 0.08 | 0.08 |  |
|  |  |  |  |  |
|  | Factor Correlations | | |  |
|  | I | II | III |  |
| I | 1.00 | -0.08 | -0.24 |  |
| II | -0.08 | 1.00 | -0.02 |  |
| III | -0.24 | -0.02 | 1.00 |  |

*Note*. *N* = 81. Factors were not assigned labels. *h*^2^ = communalities. Factors extracted using a maximum likelihood estimation and rotated using the promax procedure. Factor loadings greater than or equal to |0.4| are in bold.
