# Supplementary material for: Personality, subjective well-being, and the serotonin 1a receptor gene in common marmosets (Callithrix jacchus)
Source: PLoS One. 2021 Aug 9;16(8):e0238663. doi: 10.1371/journal.pone.0238663 (PMC8351977; doi:10.1371/journal.pone.0238663)
Supplement: S12 Table — N = 128. h2 = communalities. Factors extracted using a maximum likelihood estimation and rotated using the promax procedure. Factor loadings greater than or equal to |0.4| are in bold. (DOCX) [file pone.0238663.s026.docx]

Table S12

*Pattern Matrix from the Factor Analysis of Weighted Correlation Matrix (***R***_w_)*

|  | Factor | | |  |
| --- | --- | --- | --- | --- |
| Item | Pro-sociality | Impulsiveness | Boldness | *h*^2^ |
| Friendly | **0.82** | -0.06 | -0.01 | 0.71 |
| Aggressive | **-0.79** | 0.00 | **0.40** | 0.72 |
| Dominant | **-0.78** | -0.02 | **0.42** | 0.69 |
| Gentle | **0.75** | -0.19 | -0.01 | 0.71 |
| Sociable | **0.73** | -0.10 | 0.26 | 0.70 |
| Bullying | **-0.70** | 0.04 | 0.32 | 0.57 |
| Affectionate | **0.70** | -0.08 | 0.06 | 0.56 |
| Sympathetic | **0.70** | -0.05 | 0.11 | 0.55 |
| Stingy/greedy | **-0.66** | 0.13 | 0.29 | 0.58 |
| Defiant | **-0.66** | 0.06 | 0.39 | 0.57 |
| Helpful | **0.65** | -0.05 | 0.26 | 0.56 |
| Protective | **0.64** | -0.01 | 0.25 | 0.51 |
| Jealous | **-0.59** | 0.12 | 0.30 | 0.48 |
| Independent | **-0.58** | -0.06 | -0.10 | 0.33 |
| Dependent/follower | **0.56** | 0.03 | 0.05 | 0.31 |
| Solitary | **-0.55** | -0.03 | **-0.45** | 0.55 |
| Irritable | **-0.54** | 0.30 | 0.20 | 0.55 |
| Submissive | **0.54** | -0.09 | -0.35 | 0.42 |
| Imitative | **0.51** | 0.08 | 0.20 | 0.30 |
| Individualistic | **-0.50** | 0.14 | -0.20 | 0.39 |
| Sensitive | **0.45** | -0.25 | 0.20 | 0.41 |
| Impulsive | -0.12 | **0.68** | -0.09 | 0.54 |
| Reckless | -0.11 | **0.63** | 0.06 | 0.47 |
| Cool | 0.23 | **-0.61** | 0.01 | 0.55 |
| Thoughtless | -0.01 | **0.61** | 0.13 | 0.41 |
| Distractible | 0.02 | **0.60** | -0.03 | 0.34 |
| Unemotional | -0.04 | **-0.59** | -0.04 | 0.33 |
| Disorganized | -0.16 | **0.47** | -0.05 | 0.31 |
| Excitable | -0.33 | **0.47** | 0.08 | 0.48 |
| Stable | 0.23 | **-0.45** | 0.33 | 0.44 |
| Predictable | 0.10 | **-0.45** | 0.07 | 0.24 |
| Conventional | 0.35 | -0.37 | -0.02 | 0.38 |
| Erratic | -0.31 | 0.36 | -0.15 | 0.35 |
| Manipulative | -0.37 | -0.21 | **0.59** | 0.39 |
| Timid | 0.06 | 0.33 | **-0.58** | 0.38 |
| Active | 0.10 | **0.51** | **0.52** | 0.56 |
| Depressed | -0.12 | -0.12 | **-0.51** | 0.31 |
| Curious | 0.18 | 0.31 | **0.49** | 0.37 |
| Lazy | -0.04 | -0.36 | **-0.49** | 0.40 |
| Vulnerable | 0.22 | 0.10 | **-0.45** | 0.21 |
| Playful | 0.39 | **0.40** | **0.43** | 0.44 |
| Clumsy | -0.01 | 0.12 | **-0.42** | 0.18 |
| Inquisitive | 0.19 | 0.22 | **0.42** | 0.27 |
| Fearful | -0.02 | 0.23 | -0.38 | 0.19 |
| Inventive | 0.20 | 0.13 | 0.38 | 0.21 |
| Autistic | 0.17 | 0.09 | -0.33 | 0.12 |
| Intelligent | 0.27 | -0.23 | 0.32 | 0.29 |
| Cautious | 0.05 | -0.20 | -0.25 | 0.12 |
| Proportion of variance | 0.22 | 0.11 | 0.10 |  |
|  |  |  |  |  |
|  | Factor Correlations | | |  |
|  | Pro-sociality | Impulsiveness | Boldness |  |
| Pro-sociality | 1.00 |  |  |  |
| Impulsiveness | -0.44 | 1.00 |  |  |
| Boldness | 0.11 | 0.11 | 1.00 |  |

*Note*. *N* = 128. *h*^2^ = communalities. Factors extracted using a maximum likelihood estimation and rotated using the promax procedure. Factor loadings greater than or equal to |0.4| are in bold.
