# Supplementary material for: Personality, subjective well-being, and the serotonin 1a receptor gene in common marmosets (Callithrix jacchus)
Source: PLoS One. 2021 Aug 9;16(8):e0238663. doi: 10.1371/journal.pone.0238663 (PMC8351977; doi:10.1371/journal.pone.0238663)
Supplement: S14 Table — N = 128. h2 = communalities. Factors extracted using a maximum likelihood estimation and rotated using the promax procedure. Factor loadings greater than or equal to |0.4| are in bold. Factor correlation = -0.08. (DOCX) [file pone.0238663.s028.docx]

Table S14

*Pattern Matrix from Second-Order Factor Analysis from the Weighted Correlation Matrix (***R***_w_)*

|  | Second-order factor | |  |
| --- | --- | --- | --- |
| First-order factor | Pro-sociality | Boldness | *h*^2^ |
| Sociability | **0.79** | 0.25 | 0.66 |
| Dominance | **-0.70** | 0.24 | 0.57 |
| Impulsiveness | **-0.66** | 0.01 | 0.44 |
| Negative affect | 0.04 | **-0.75** | 0.56 |
| Openness | 0.03 | **0.53** | 0.28 |
| Proportion of variance | 0.31 | 0.19 |  |

*Note*. *N* = 128. *h*^2^ = communalities. Factors extracted using a maximum likelihood estimation and rotated using the promax procedure. Factor loadings greater than or equal to |0.4| are in bold. Factor correlation = -0.08.
