# Supplementary material for: Personality, subjective well-being, and the serotonin 1a receptor gene in common marmosets (Callithrix jacchus)
Source: PLoS One. 2021 Aug 9;16(8):e0238663. doi: 10.1371/journal.pone.0238663 (PMC8351977; doi:10.1371/journal.pone.0238663)
Supplement: S15 Table — N = 122. (DOCX) [file pone.0238663.s029.docx]

Table S15

*Effects of G840C Genotype on Personality Domains*

|  | *b* | *SE* | *t* | *P* |
| --- | --- | --- | --- | --- |
| Sociability |  |  |  |  |
| Intercept | 0.85 | 0.30 | 2.81 | 0.006 |
| Male vs. Female | -0.75 | 0.22 | -3.49 | < 0.001 |
| Age | -0.01 | 0.03 | -0.34 | 0.74 |
| GC vs. CC | -0.22 | 0.23 | -0.95 | 0.35 |
| GG vs. CC | -0.33 | 0.27 | -1.21 | 0.23 |
| Dominance |  |  |  |  |
| Intercept | -0.94 | 0.30 | -3.08 | 0.003 |
| Male vs. Female | 0.70 | 0.22 | 3.23 | 0.002 |
| Age | -0.01 | 0.03 | -0.20 | 0.84 |
| GC vs. CC | 0.51 | 0.23 | 2.19 | 0.03 |
| GG vs. CC | 0.58 | 0.27 | 2.12 | 0.036 |
| Impulsiveness |  |  |  |  |
| Intercept | -0.59 | 0.30 | -1.96 | 0.052 |
| Male vs. Female | 0.65 | 0.22 | 3.01 | 0.003 |
| Age | -0.02 | 0.03 | -0.68 | 0.50 |
| GC vs. CC | 0.17 | 0.23 | 0.73 | 0.47 |
| GG vs. CC | 0.37 | 0.27 | 1.35 | 0.18 |
| Openness |  |  |  |  |
| Intercept | -0.08 | 0.30 | -0.26 | 0.80 |
| Male vs. Female | 0.45 | 0.22 | 2.08 | 0.04 |
| Age | -0.05 | 0.03 | -1.41 | 0.16 |
| GC vs. CC | -0.03 | 0.23 | -0.12 | 0.91 |
| GG vs. CC | -0.09 | 0.27 | -0.31 | 0.75 |
| Negative Affect |  |  |  |  |
| Intercept | 0.48 | 0.32 | 1.50 | 0.14 |
| Male vs. Female | -0.14 | 0.23 | -0.63 | 0.53 |
| Age | -0.06 | 0.03 | -1.60 | 0.11 |
| GC vs. CC | -0.10 | 0.24 | -0.40 | 0.69 |
| GG vs. CC | -0.16 | 0.29 | -0.55 | 0.58 |
| Pro-sociality |  |  |  |  |
| Intercept | 0.94 | 0.30 | 3.16 | 0.002 |
| Male vs. Female | -0.81 | 0.21 | -3.83 | < 0.001 |
| Age | 0.01 | 0.03 | 0.18 | 0.86 |
| GC vs. CC | -0.37 | 0.23 | -1.63 | 0.11 |
| GG vs. CC | -0.51 | 0.27 | -1.90 | 0.06 |
| Boldness |  |  |  |  |
| Intercept | -0.32 | 0.31 | -1.01 | 0.31 |
| Male vs. Female | 0.36 | 0.22 | 1.63 | 0.11 |
| Age | 0.00 | 0.03 | 0.05 | 0.96 |
| GC vs. CC | 0.04 | 0.24 | 0.16 | 0.88 |
| GG vs. CC | 0.03 | 0.28 | 0.12 | 0.90 |

*Note*. *N* = 122.
