# Supplementary material for: Personality, subjective well-being, and the serotonin 1a receptor gene in common marmosets (Callithrix jacchus)
Source: PLoS One. 2021 Aug 9;16(8):e0238663. doi: 10.1371/journal.pone.0238663 (PMC8351977; doi:10.1371/journal.pone.0238663)
Supplement: S16 Table — N = 122. (DOCX) [file pone.0238663.s030.docx]

Table S16

*Effects of G840C Genotype on Personality Domains*

|  | Genotype | | | | | | | | |
| --- | --- | --- | --- | --- | --- | --- | --- | --- | --- |
|  | C Present vs. Absent | | | |  | G Present vs. Absent | | | |
|  | *b* | *SE* | *t* | *P* |  | *b* | *SE* | *t* | *P* |
| Sociability |  |  |  |  |  |  |  |  |  |
| Intercept | 0.49 | 0.28 | 1.76 | 0.082 |  | 0.86 | 0.30 | 2.87 | 0.005 |
| Male vs. Female | -0.74 | 0.21 | -3.43 | < 0.001 |  | -0.75 | 0.21 | -3.50 | < 0.001 |
| Age | -0.01 | 0.03 | -0.23 | 0.82 |  | -0.01 | 0.03 | -0.40 | 0.69 |
| Genotype | 0.17 | 0.21 | 0.79 | 0.43 |  | -0.25 | 0.22 | -1.13 | 0.26 |
| Impulsiveness |  |  |  |  |  |  |  |  |  |
| Intercept | -0.28 | 0.29 | -1.00 | 0.32 |  | -0.94 | 0.30 | -3.12 | 0.002 |
| Male vs. Female | 0.66 | 0.22 | 3.02 | 0.003 |  | 0.70 | 0.22 | 3.24 | 0.002 |
| Age | -0.01 | 0.03 | -0.44 | 0.66 |  | -0.01 | 0.03 | -0.16 | 0.87 |
| Genotype | -0.20 | 0.21 | -0.93 | 0.36 |  | 0.53 | 0.22 | 2.37 | 0.019 |
| Dominance |  |  |  |  |  |  |  |  |  |
| Intercept | -0.20 | 0.28 | -0.72 | 0.47 |  | -0.61 | 0.30 | -2.03 | 0.044 |
| Male vs. Female | 0.64 | 0.21 | 2.97 | 0.004 |  | 0.65 | 0.22 | 3.01 | 0.003 |
| Age | -0.03 | 0.03 | -0.77 | 0.44 |  | -0.02 | 0.03 | -0.57 | 0.57 |
| Genotype | -0.24 | 0.21 | -1.16 | 0.25 |  | 0.23 | 0.22 | 1.01 | 0.31 |
| Openness |  |  |  |  |  |  |  |  |  |
| Intercept | -0.17 | 0.28 | -0.60 | 0.55 |  | -0.07 | 0.30 | -0.24 | 0.81 |
| Male vs. Female | 0.45 | 0.21 | 2.10 | 0.038 |  | 0.45 | 0.22 | 2.09 | 0.039 |
| Age | -0.05 | 0.03 | -1.41 | 0.16 |  | -0.05 | 0.03 | -1.46 | 0.15 |
| Genotype | 0.07 | 0.21 | 0.31 | 0.76 |  | -0.04 | 0.22 | -0.20 | 0.84 |
| Negative Affect |  |  |  |  |  |  |  |  |  |
| Intercept | 0.31 | 0.29 | 1.04 | 0.30 |  | 0.48 | 0.32 | 1.53 | 0.13 |
| Male vs. Female | -0.14 | 0.23 | -0.60 | 0.55 |  | -0.14 | 0.23 | -0.63 | 0.53 |
| Age | -0.05 | 0.03 | -1.57 | 0.12 |  | -0.06 | 0.03 | -1.65 | 0.10 |
| Genotype | 0.09 | 0.22 | 0.39 | 0.70 |  | -0.12 | 0.23 | -0.49 | 0.62 |
| Pro-sociality |  |  |  |  |  |  |  |  |  |
| Intercept | 0.38 | 0.28 | 1.38 | 0.17 |  | 0.96 | 0.30 | 3.22 | 0.002 |
| Male vs. Female | -0.79 | 0.21 | -3.69 | < 0.001 |  | -0.81 | 0.21 | -3.84 | < 0.001 |
| Age | 0.01 | 0.03 | 0.37 | 0.72 |  | 0.00 | 0.03 | 0.10 | 0.92 |
| Genotype | 0.23 | 0.21 | 1.11 | 0.27 |  | -0.41 | 0.22 | -1.87 | 0.064 |
| Boldness |  |  |  |  |  |  |  |  |  |
| Intercept | -0.28 | 0.29 | -0.96 | 0.34 |  | -0.32 | 0.31 | -1.02 | 0.31 |
| Male vs. Female | 0.36 | 0.22 | 1.63 | 0.11 |  | 0.36 | 0.22 | 1.64 | 0.10 |
| Age | 0.00 | 0.03 | 0.03 | 0.97 |  | 0.00 | 0.03 | 0.05 | 0.96 |
| Genotype | -0.01 | 0.22 | -0.03 | 0.98 |  | 0.04 | 0.23 | 0.16 | 0.87 |

*Note*. *N* = 122.
