# Supplementary material for: Personality, subjective well-being, and the serotonin 1a receptor gene in common marmosets (Callithrix jacchus)
Source: PLoS One. 2021 Aug 9;16(8):e0238663. doi: 10.1371/journal.pone.0238663 (PMC8351977; doi:10.1371/journal.pone.0238663)
Supplement: S17 Table — N = 123. (DOCX) [file pone.0238663.s031.docx]

Table S17

*Effects of T901A Genotype on Personality Domains*

|  | *b* | *SE* | *t* | *P* |
| --- | --- | --- | --- | --- |
| Sociability |  |  |  |  |
| Intercept | 0.73 | 0.24 | 3.12 | 0.002 |
| Male vs. Female | -0.72 | 0.21 | -3.43 | < 0.001 |
| Age | 0.01 | 0.03 | 0.17 | 0.87 |
| T allele present | -0.26 | 0.19 | -1.39 | 0.17 |
| Impulsiveness |  |  |  |  |
| Intercept | -0.59 | 0.23 | -2.50 | 0.014 |
| Male vs. Female | 0.64 | 0.21 | 3.07 | 0.003 |
| Age | -0.03 | 0.03 | -0.89 | 0.38 |
| T allele present | 0.29 | 0.19 | 1.54 | 0.13 |
| Dominance |  |  |  |  |
| Intercept | -0.37 | 0.23 | -1.58 | 0.12 |
| Male vs. Female | 0.64 | 0.21 | 3.07 | 0.003 |
| Age | -0.04 | 0.03 | -1.19 | 0.24 |
| T allele present | 0.05 | 0.19 | 0.27 | 0.79 |
| Openness |  |  |  |  |
| Intercept | -0.09 | 0.25 | -0.36 | 0.72 |
| Male vs. Female | 0.59 | 0.22 | 2.67 | 0.009 |
| Age | -0.05 | 0.03 | -1.34 | 0.18 |
| T allele present | -0.20 | 0.20 | -0.99 | 0.32 |
| Negative Affect |  |  |  |  |
| Intercept | 0.36 | 0.25 | 1.44 | 0.15 |
| Male vs. Female | -0.15 | 0.22 | -0.67 | 0.5 |
| Age | -0.05 | 0.03 | -1.49 | 0.14 |
| T allele present | 0.02 | 0.20 | 0.09 | 0.93 |
| Pro-sociality |  |  |  |  |
| Intercept | 0.67 | 0.23 | 2.92 | 0.004 |
| Male vs. Female | -0.77 | 0.20 | -3.79 | < 0.001 |
| Age | 0.03 | 0.03 | 0.87 | 0.39 |
| T allele present | -0.25 | 0.19 | -1.36 | 0.18 |
| Boldness |  |  |  |  |
| Intercept | -0.26 | 0.25 | -1.03 | 0.30 |
| Male vs. Female | 0.45 | 0.22 | 2.04 | 0.044 |
| Age | 0.00 | 0.03 | 0.00 | > 0.99 |
| T allele present | -0.13 | 0.20 | -0.67 | 0.51 |

*Note*. *N* = 123.
